# Comparative study on antimicrobial activity of mono-rhamnolipid and di-rhamnolipid and exploration of cost-effective antimicrobial agents for agricultural applications

**DOI:** 10.1186/s12934-022-01950-x

**Published:** 2022-10-23

**Authors:** Feng Zhao, Bingxin Wang, Menglin Yuan, Sijia Ren

**Affiliations:** School of Life Sciences, Qufu Normal University, Qufu, 273165 Shandong Province China

**Keywords:** Biosurfactants, Antimicrobial activity, Biocontrol, *Pseudomonas aeruginosa*, Mono-rhamnolipid

## Abstract

**Background:**

Chemical pesticides have defects in crop diseases control, such as narrow antimicrobial spectrum, chemicals residue risk and harm to farmland ecosystem. Antimicrobial agents from microbial sources are highly interested in agriculture. Studies showed that rhamnolipid biosurfactants possessed certain antimicrobial activity. The structural differences in rhamnolipid inevitably affect their activities. But the antimicrobial effect of mono-rhamnolipid and di-rhamnolipid is unknown. Rhamnolipid with unique structure can be produced using specific microbial cell factory.

**Results:**

Different types of rhamnolipid were produced from different *Pseudomonas aeruginosa* strains. Rha-C_10_-C_10_ and Rha-Rha-C_10_-C_10_ were the main homologues in the separated mono-rhamnolipid and di-rhamnolipid, respectively. Both mono-rhamnolipid and di-rhamnolipid exhibited certain antimicrobial activity against the tested microbial strains, especially the fungi and Gram-positive bacteria. But mono-rhamnolipid was superior to di-rhamnolipid, with inhibition zone diameters larger than 25 mm and inhibition rate higher than 90%. The IC50 values of mono-rhamnolipid were lower than 5 mg/L against the tested bacterium and fungus, whereas the IC50 values of di-rhamnolipid were ranged from 10 mg/L to 20 mg/L. Mono-rhamnolipid stimulated the tested strains to generate higher level of intracellular ROS. Mono-rhamnolipid exhibited better antimicrobial activity to the potential agricultural pathogens, such as *Alternaria alternata*, *Pantoea agglomerans* and *Cladosporium* sp. The mono-rhamnolipid crude extract of strain *P*. *aeruginosa* SGΔrhlC can replace the separated mono-rhamnolipid. After 50 times dilution, the fermentation broth of the mono-rhamnolipid producing strain SGΔrhlC exhibited equal antimicrobial effect to mono-rhamnolipid (200 mg/L). Prospects of mono-rhamnolipid were also discussed for antimicrobial applications in agriculture.

**Conclusions:**

This work discovered that mono-rhamnolipid was superior to di-rhamnolipid on antimicrobial activity for agricultural applications. Mono-rhamnolipid is an excellent candidate for agricultural biocontrol. The knockout strain *P. aeruginosa* SGΔrhlC is an excellent microbial cell factory for high producing mono-rhamnolipid. Its mono-rhamnolipid crude extract and its diluted fermentation broth are cost-effective antimicrobial agents. This work provided new insights to develop green and efficient antimicrobial agents for agricultural applications.

## Background

Plant pathogens can cause crop diseases such as wilt, rot, spots, white leaves, blight, and even make plants die [1]. Crop diseases not only threaten crop yield, but also influence the quality and safety of agricultural products [2]. Control of agricultural pathogens is of great scientific significance and application value. Chemical pesticides have defects in crop diseases control, such as narrow antimicrobial spectrum, chemicals residue risk and harm to farmland ecosystem [3–5]. The efficient and green antimicrobial agents have gradually attracted more attention.

Antimicrobial agents from microbial source are highly interested in agriculture. Biosurfactants are metabolites synthesized by microorganisms, including glycolipids and lipopeptides [6–8]. Due to their amphiphilic molecular structure, biosurfactants possess antibacterial, emulsifying, solubilizing and osmotic activities [6]. Biosurfactants can control soil-borne diseases and has a wide antimicrobial spectrum [7, 8]. Studies have also shown that biosurfactants can enhance the plants immunity and improve the fertilizer utilization efficiency [9, 10]. Using biosurfactants to control agricultural pathogens is green and eco-friendly, and it also accords with the development direction of ecological agriculture [7, 8].

Among biosurfactants, rhamnolipid has been widely studied due to its relatively high yield and good activity [11, 12]. Rhamnolipid is mainly produced by *Pseudomonas aeruginosa*. Studies showed that rhamnolipid possessesed certain antibacterial activity. The molecular structure of rhamnolipid is diverse [13]. The structural differences in rhamnolipid inevitably affect their activities [13, 14]. Rhamnolipids can be divided into mono-rhamnolipid and di-rhamnolipid according to the contained rhamnosyl number [15]. But the antimicrobial effect of mono-rhamnolipid and di-rhamnolipid is unknown.

This study aims to explore green and cost-effective agricultural antimicrobial agents from microbial sources. In the present study, different types of rhamnolipid were produced from different *P. aeruginosa* strains. The mono-rhamnolipid and di-rhamnolipid were separated. The antimicrobial activity of mono-rhamnolipid and di-rhamnolipid were compared by agar diffusion method, turbidimetric method and IC50 assay. Rhamnolipid with specific structure and high antibacterial activity was screened. The antimicrobial mechanism of mono-rhamnolipid and di-rhamnolipid was studied and discussed by detecting intracellular Reactive Oxygen Species (ROS). The knockout strain *P. aeruginosa* SGΔrhlC was chosen for high producing mono-rhamnolipid. The crude extract of rhamnolipid and the dilution of rhamnolipid fermentation broth were attempted for antimicrobial evaluation to explore the economical antimicrobial agents. The results will provide new insights to explore green and efficient agricultural antimicrobial agents from microbial sources.

## Materials and methods

### Strains and culture medium

Strain *P. aeruginosa* SG was used to produce the conventional rhamnolipids containing mono-rhamnolipid and di-rhamnolipid [16]. The knockout strain *P. aeruginosa* SGΔrhlC was used for producing only mono-rhamnolipid [17]. The seed culture of strain SG and strain SGΔrhlC was prepared using LB medium at 35 °C and 180 rpm. The amount of inoculum for fermentation was 3% (v/v). The medium for rhamnolipid production contained 45 g/L glycerol, 3.5 g/L NaNO3, 4.0 g/L K2HPO4·3H2O, 3.0 g/L KH2PO4, 1.0 g/L MgSO_4_·7H2O. The pH of medium was adjusted to 6.8 using the 2 mol/L NaOH solution. Rhamnolipid production by strain SG and strain SGΔrhlC was performed at 35 °C and 180 rpm for 5 days. In antimicrobial experiments, the tested bacteria were *Escherichia coli* DH5α, *Bacillus wiedmannii* H238, *B. Safensis* B36# and *Pantoea agglomerans* B10. The tested fungi were *Alternaria alternata* G2, *Cladosporium* sp. B, *Actinomucor* sp. Y and *Penicillium oxalicum* S11. LB medium and LB agar plate medium were used as culture media for the tested bacteria. Potato Dextrose Broth (PDB) and Potato Dextrose Agar (PDA) plate medium were used for the tested fungi.

### Analytical methods for antimicrobial activity evaluation

The agar diffusion method was used to evaluate the antimicrobial activity of samples against the tested strains on solid culture medium. LB medium and PDB medium were used to prepare batch culture of tested bacteria and fungi, respectively. The batch culture was diluted 10^5^ times and then coated on LB agar plates medium and PDA agar plates medium, respectively. The sterile filter papers with a diameter of 6 mm were placed on the plates. Then 10 μL of antimicrobial agents were added to each filter paper. The culture condition for the tested bacteria was 35 °C for 1 day, and the culture condition for the tested fungi was 28 °C for 2 days. After culture, the diameters of inhibition zone around the filter papers were measured. Measurements are accurate to 0.5 mm. The single factor analysis of variance (ANOVA) was performed to compare the results. The turbidimetric method was used to investigate the antimicrobial activity of samples against some of the tested strains in liquid culture. Biomass of strains was represented by the OD_600_ values of their culture. The batch culture of tested bacteria and fungi was inoculated into tubes containing 8 mL LB medium or PDB medium, respectively. The inoculum amount was 1% (v/v). Antimicrobial agents were added into test tubes to a certain concentration. The liquid culture conditions for the tested bacteria were 35 °C and 180 rpm for 1 day, and the liquid culture conditions for the tested fungi were 28 °C and 180 rpm for 2 days. After culture, the OD_600_ values of culture were determined by UV spectrophotometer. Based on the OD_600_ values, the inhibition rate (%) of the experimental group was calculated compared with the control group.

### Extraction of rhamnolipid from culture of strain SG and SGΔrhlC

The crude extract of rhamnolipid was obtained referring to the method previously described with minor modifications [14, 18]. Firstly, the culture broth was centrifuged at 8000 g for 10 min to remove insoluble substances and bacterial cells. The supernatant was heated in the water bath at 80 °C for 15 min to denature the soluble protein in the broth. The supernatant was collected by centrifugation at 8000 g for 10 min. The pH of supernatant was adjusted to 2.0 using 6 mol/L HCl solution. The supernatant was kept at 4 °C for 8 h. The precipitation was collected by centrifugation at 8000 g for 10 min. The precipitation was dissolved in methanol, and then the organic phase was collected by centrifugation at 8000 g for 5 min. The solid crude extract of rhamnolipid was obtained by vacuum freeze-drying.

### Separation of mono-rhamnolipid and di-rhamnolipid and HPLC–MS validation

For further study, mono-rhamnolipid (Mono-RL) and di-rhamnolipid (Di-RL) were separated from rhamnolipid products of strain *P. aeruginosa* SG using silica gel column chromatography. Based on the separation procedures previously described [18–20], the crude extract of rhamnolipid was dissolved in chloroform and loaded on top of the silica gel column. The column was washed by chloroform (100%) to remove the neutral lipids and other impurities. Then the column was gradually eluted by mobile phases of chloroform/methanol at 2:1 (v/v), 1:1 (v/v), 1:2 (v/v). Every 15 mL eluted sample was collected using test tubes. Thin-layer chromatography (TLC) was used to detect Mono-RL and Di-RL with a mobile phase of chloroform/methanol/H_2_O (65:7:2). Mono-RL was eluted first, and then Di-RL. The eluted solution of Mono-RL and Di-RL was respectively combined. The solvent was removed by vacuum rotary evaporator at 50℃ and 50 rpm. Finally, the separated Mono-RL and Di-RL was validated by HPLC–MS analysis. The HPLC–MS analysis was referred to the previous studies [14, 21]. Briefly, the separated Mono-RL and Di-RL were dissolved into 10% acetonitrile water solution with rhamnolipid concentrations of 500 mg/L. The acetonitrile–water gradient from 10 to 60% was used as mobile phase. The C18 reversed phase column was used. The detection wavelength was 220 nm (UV). The sample size for HPLC was 20 μL. The flow rate was 0.6 mL/min. For the mass spectrometer, the capillary voltage was 3.8 kV, and ion source temperature was 120 °C. The negative ion mode was chosen. The scanning mass number were ranged from 50 m/Z to 1000 m/Z.

### Comparison on antimicrobial activity of mono-rhamnolipid and di-rhamnolipid

The antimicrobial activity of mono-rhamnolipid (Mono-RL) and di-rhamnolipid (Di-RL) was compared. As described in 2.2., agar diffusion method and turbidimetric method were used to evaluate the antimicrobial activity of Mono-RL and Di-RL. The tested bacterial strains were *E. coli* DH5α, *B. wiedmannii* H238, *B. Safensis* B36# and *P. agglomerans* B10. The tested fungi strains were *A. alternata* G2, *Cladosporium* sp. B, *Actinomucor* sp. Y and *P. oxalicum* S11. Mono-RL and Di-RL were used at a concentration of 200 mg/L. After solid culture, the diameters (mm) of inhibition zone formed by Mono-RL and Di-RL were recorded. After liquid culture, the inhibition rates (%) of Mono-RL and Di-RL were calculated compared with the control group, based on the OD_600_ values.

### The IC50 estimation of mono-rhamnolipid and di-rhamnolipid

The 50% inhibiting concentration (IC50) is another evaluation parameter to characterize the antibacterial activity of antimicrobial agents. IC50 refers to the required agent concentration when half of pathogen is inhibited [22]. Mono-RL and Di-RL were used as antimicrobial agents. *B. Wiedmannii* H238 and *A. alternata* G2 were used as the test strains. The IC50 values of Mono-RL and Di-RL were determined by the OD_600_ changes in liquid culture. The determination method of IC50 is briefly described as follows. The batch culture of the tested strains was inoculated into test tubes containing LB medium or PDB medium, respectively. The inoculum amount was 1% (v/v). Then the tubes without any rhamnolipid were set as control group, and tubes adding with different concentrations of rhamnolipid were set as experimental groups. In the experimental groups, the concentrations of rhamnolipid were 5 mg/L, 10 mg/L, 15 mg/L, 25 mg/L and 50 mg/L. Each group performed in triplicate. After culture, the OD_600_ in each test tube was measured by UV spectrophotometer. The inhibition curves of rhamnolipid were prepared. According to the inhibition curves, the concentration of rhamnolipid in the experimental group when OD_600_ was half of that in the control group was estimated, namely, its IC50 value.

### Reactive oxygen species detection

The antimicrobial mechanism of mono-rhamnolipid and di-rhamnolipid was studied and discussed by detecting intracellular Reactive Oxygen Species (ROS). Reactive Oxygen Species Assay Kit S0033S (Beyotime Biotechnology, Shanghai, China) was used in this study. Mono-rhamnolipid and di-rhamnolipid were used as irritant. The tested microbial strains were *B. wiedmannii* H238 and *A. alternata* G2. According to the manufacturer’s instruction, the collected microbial cells were incubated in DCFH-DA solution at 35 °C for 20 min. The fluorescence probe DCFH-DA were loaded into microbial cells. Then treated microbial cells were stimulated to produce ROS by Mono-RL and Di-RL at 35 °C for 3 h. DMSO was used as control. Intracellular ROS can oxidize non-fluorescent DCFH to fluorescent DCF. The intensity of fluorescence was measured at the excitation wavelength of 488 nm and emission wavelength of 525 nm using a microplate reader. The fluorescence value was used as an indirect indicator of intracellular ROS level.

### Exploration of economical antimicrobial agents

Mono-rhamnolipid is promising for agricultural biocontrol. Separation and purification of mono-rhamnolipid is too complex and high-cost. How to further reduce the cost in application? In order to explore economical antimicrobial agents, the crude extracts of rhamnolipid and the fermentation broth of rhamnolipid were used for further antimicrobial evaluation.

The rhamnolipid crude extract R1 from the knockout strain SGΔrhlC and rhamnolipid crude extract R2 from wild-type strain SG were used as antimicrobial agents. The antimicrobial activity of R1 and R2 were studied by agar diffusion method as described in 2.2. Here, *B. wiedmannii* H238, *P. agglomerans* B10, *A. alternata* G2 and *Cladosporium* sp. B were used as the test strains. R1 and R2 were used at a concentration of 200 mg/L. After solid culture, the diameters (mm) of inhibition zone formed by R1 and R2 were measured recorded.

The rhamnolipid fermentation broth of the knockout strain SGΔrhlC and wild-type strain SG were used as antimicrobial agents. The antimicrobial activity of rhamnolipid fermentation broth with different dilution ratio was evaluated by turbidimetric method as described in 2.2. The test strains were *E. coli* DH5α, *B. wiedmannii* H238, *B. Safensis* B36#, *P. agglomerans* B10, *A. alternata* G2 and *P. oxalicum* S11. The dilution ratios of rhamnolipid fermentation broth were 10 times, 20 times and 50 times. The tubes without any rhamnolipid were set as negative control group. The tubes adding with 200 mg/L of rhamnolipid crude extracts R1 or R2 were set as positive groups. Each group performed in triplicate. After culture, the OD_600_ in each test tube was measured by UV spectrophotometer. Compared with the negative control group, the inhibition rates (%) were calculated based on the OD_600_ values. The inhibition rates (%) of two kinds of rhamnolipid fermentation broth were compared with their crude extracts of rhamnolipid, R1 and R2.

## Results and discussion

### HPLC–MS analysis of separated mono-rhamnolipid and di-rhamnolipid

**Fig. 1:**
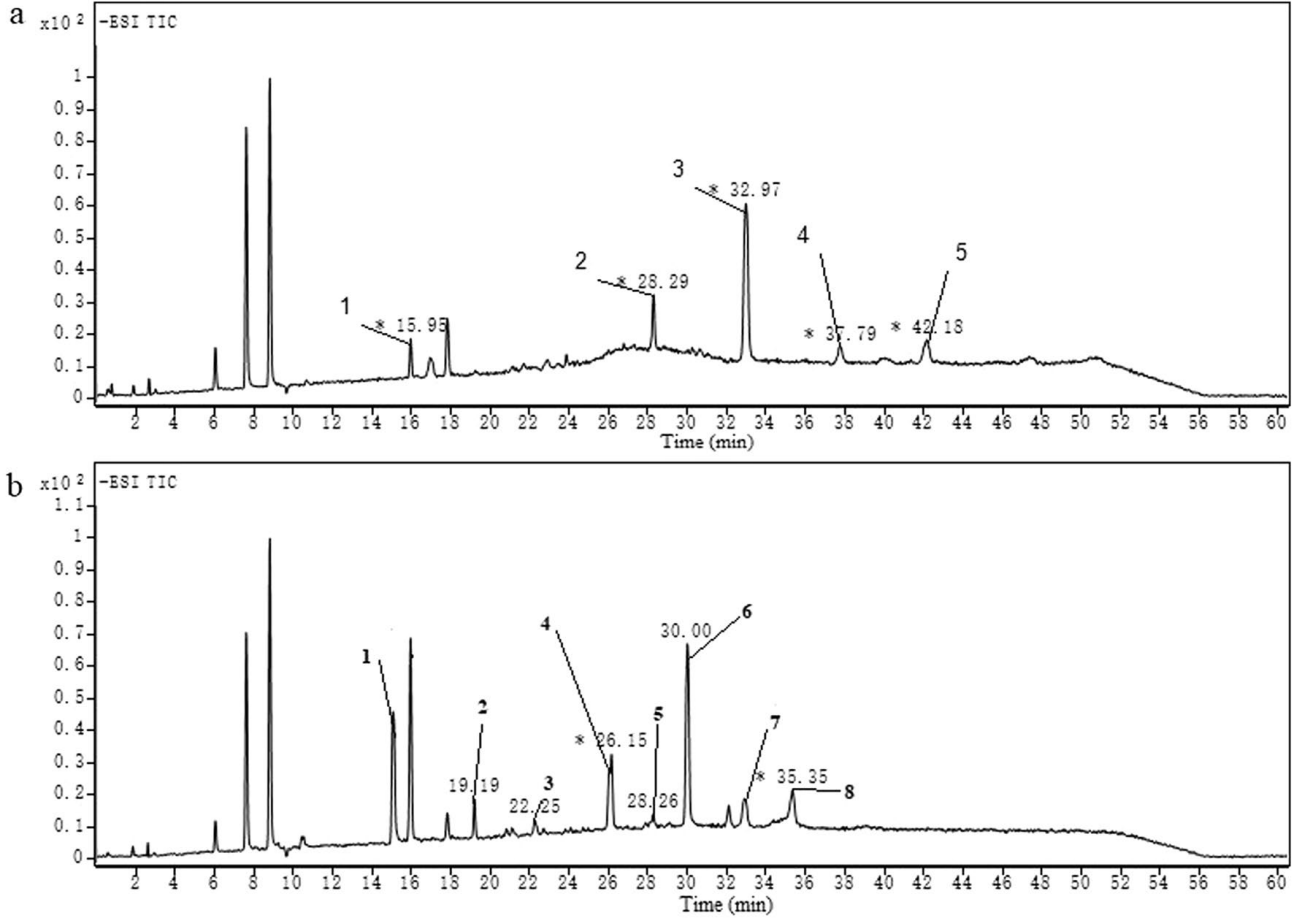
Liquid chromatogram results of the separated rhamnolipids: **a** mono-rhamnolipid, **b** di-rhamnolipid

Structural compositions of the separated mono-rhamnolipid and di-rhamnolipid was validated by HPLC–MS. The liquid chromatogram results of the separated mono-rhamnolipid and di-rhamnolipid as shown in Fig. 1. Based on the m/z analysis method previously described [14, 21], the rhamnolipid homologues identified in the separated mono-rhamnolipid and di-rhamnolipid were listed in Table 1. There were 5 kinds of mono-rhamnolipid homologues but no di-rhamnolipid homologues in the separated mono-rhamnolipid. The separated di-rhamnolipid contained 7 kinds of di-rhamnolipid homologues but no mono-rhamnolipid homologues. Results demonstrated that mono-rhamnolipid and di-rhamnolipid were successfully separated from conventional rhamnolipid products. Based on the peak area, Rha-C_10_-C_10_ was the main homologues in the separated mono-rhamnolipid, and Rha-Rha-C_10_-C_10_ was the main homologues in the separated di-rhamnolipid. Provious studies also reported that Rha-C_10_-C_10_ and Rha-Rha-C_10_-C_10_ were the main homologues in rhamnolipid produced by *P. aeruginosa* [20, 23].

**Table 1:** Structural compositions of the separated mono-rhamnolipid and di-rhamnolipid

Chromatographic peak number	Retention time (min)	Mass spectrum signal (m/z)	Rhamnolipid homologues
The separated mono-rhamnolipid component			
1	15.95	333	Rha-C10
2	28.29	475	Rha-C8-C10
3	32.97	503	Rha-C10-C10
4	37.79	529	Rha-C10-C12:1
5	42.18	531	Rha-C10-C12
The separated di-rhamnolipid component			
1	15.05	479	Rha-Rha-C10
2	19.19	507	Rha-Rha-C12
3	22.25	593	Rha-Rha-C8-C8
4	26.15	621	Rha-Rha-C8-C10
5	28.26	647	Rha-Rha-C8-C12:1
6	30.00	649	Rha-Rha-C10-C10
7	32.90	675	Rha-Rha-C10-C12:1
8	35.35	677	Rha-Rha-C10-C12

### Antimicrobial activity of mono-rhamnolipid and di-rhamnolipid

The results of inhibition zone diameters formed by mono-rhamnolipid (Mono-RL) and di-rhamnolipid (Di-RL) on solid culture medium were shown in Table 2. Both Mono-RL and Di-RL had certain antimicrobial activity against the tested strains, especially the fungi and Gram-positive bacteria. Both Mono-RL and Di-RL showed weak antimicrobial activity against Gram-negative bacteria. This may be due to the thicker cell structure and extracellular polymers of Gram-negative bacteria, which can resist the entry of antibacterial substances into cells. So the tested Gram-negative bacteria show certain resistance to rhamnolipid. The single factor analysis of variance (ANOVA) was performed to compare the antimicrobial activity of mono-RL and di-RL. For all the tested strains, all the obtained *P* values were less than 0.05 between the antimicrobial groups of mono-RL and di-RL. Results of inhibition zone indicated that the antimicrobial effect of mono-RL was superior to that of di-RL for both bacteria and fungi. As shown in Table 3, the inhibition rate of mono-RL and di-RL against Gram-positive bacteria and fungi was higher than 90%. The inhibition rate to Gram-positive bacteria was close to 100%. Both Mono-RL and Di-RL also exhibited weak antimicrobial activity against Gram-negative bacteria. For all the tested bacteria and fungi, the antimicrobial activity of mono-RL was stronger than that of di-RL. The ANOVA analysis was also performed to compare the inhibition rates of mono-RL and di-RL. Except for the strain *A. alternata* G2, the inhibition rates of mono-RL was significantly higher than that of di-RL against the tested strains (*P* < 0.05). Results demonstrated that mono-rhamnolipid was more effective for antimicrobial applications. Studies reported that *A. alternata*, *P. agglomerans*, *Cladosporium* sp., *P. oxalicum* were potential plant pathogens in agriculture [24–26]. Mono-rhamnolipid and di-rhamnolipid exhibited antimicrobial activity to the potential agricultural pathogens such as *A. alternata*, *P. agglomerans* and *Cladosporium* sp. But mono-rhamnolipid was better. Compared with di-rhamnolipid, the hydrophilic moiety of mono-rhamnolipids contains only one rhamnose. In the momo-rhamnolipid molecule, the fatty acid chains occupy a relative larger molecular volume. So mono-rhamnolipid possesses better lipophilic properties. It was speculated that mono-rhamnolipid exhibited stronger cytolysis activity and was more likely to cause cell death of plant pathogens.

**Table 2:** Inhibition zone of mono-rhamnolipid and di-rhamnolipid against different bacteria and fungi

Strains	Inhibition zone diameters of Mono-RL (mm)	Inhibition zone diameters of Di-RL (mm)
*E. coli* DH5α	14.7 ± 1.5	6.3 ± 0.6
*B. wiedmannii* H238	30.7 ± 2.5	20.3 ± 1.5
*B. safensis* B36#	29.7 ± 1.5	19.0 ± 2.0
*P. agglomerans* B10	12.7 ± 2.1	6.7 ± 0.6
*A. alternata* G2	26.5 ± 2.3	20.2 ± 1.6
*Actinomucor* sp. Y	39.3 ± 2.1	22.7 ± 2.5
*P. oxalicum* S11	48.7 ± 2.5	23.8 ± 2.6
*Cladosporium* sp. B	35.3 ± 2.1	27.0 ± 2.0

**Table 3:** Antimicrobial activity of mono-rhamnolipid and di-rhamnolipid on microbial strains in liquid culture

Strains	Cell density (OD_600_)			Inhibition rate (%)	
	Control group	Mono-RL group	Di-RL group	Mono-RL	Di-RL
*E. coli* DH5α	2.58	2.05	2.33	20.5	9.8
*B. wiedmannii* H238	2.13	0.02	0.05	98.9	97.8
*B. safensis* B36#	2.42	0.00	0.02	100.0	99.1
*P. agglomerans* B10	2.68	1.75	2.18	34.8	18.8
*A. alternata* G2	1.93	0.10	0.16	94.6	91.9
*P. oxalicum* S11	2.23	0.16	0.22	92.8	90.3

The results showed that mono-rhamnolipid had greater application potential for agricultural biocontrol. This work discovered that mono-rhamnolipid was superior to di-rhamnolipid on agricultural antimicrobial activity. Mono-rhamnolipid has a wide antimicrobial spectrum and is microbial origin. Mono-rhamnolipid is an excellent candidate for the control of agricultural pathogens. An efficient, green and broad-spectrum agricultural antimicrobial agent is expected to be developed based on mono-rhamnolipid.

### IC50 values of mono-rhamnolipid and di-rhamnolipid

**Fig. 2:**
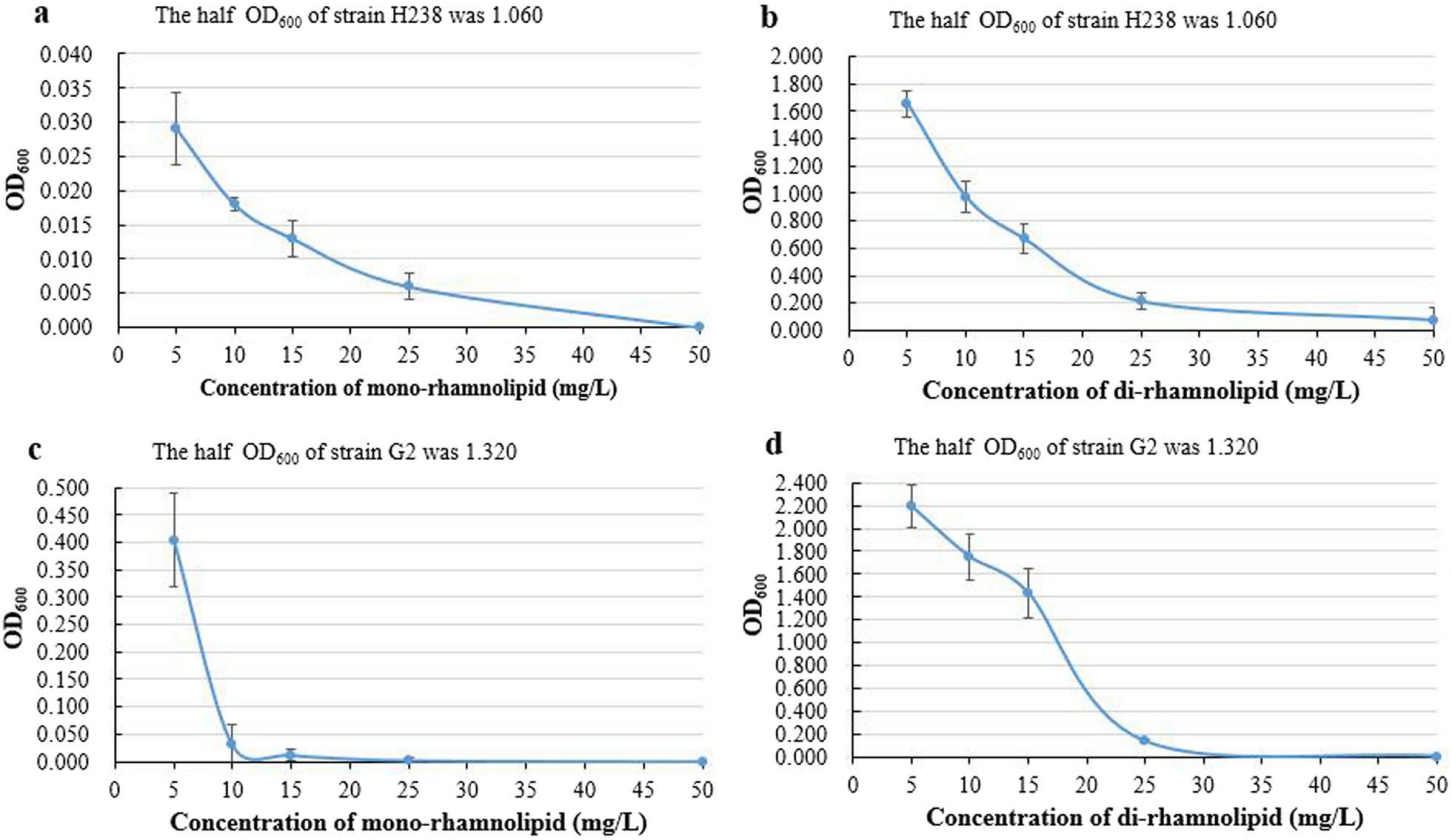
Inhibition curves of two rhamnolipid extracts at different concentrations. **a** mono-rhamnolipid against *B. wiedmannii* H238, **b** di-rhamnolipid against *B. wiedmannii* H238, **c** mono-rhamnolipid against *A. alternate* G2, **d** di-rhamnolipid against *A. alternate* G2

The inhibition curves of mono-rhamnolipid and di-rhamnolipid at different concentrations were shown in Fig. 2. The IC50 (50% inhibiting concentration) is the concentration of the inhibitor required when half of pathogen is inhibited [22]. In the control group without any rhamnolipid, the average OD_600_ value of strain *B. Wiedmannii* H238 was 2.119. So the half concentration of strain H238 was 1.060 (OD_600_ value). As shown in Fig. 2a, it can be estimated that the IC50 value of mono-rhamnolipid against strain H238 was lower than 5 mg/L, whereas the IC50 value of di-rhamnolipid against strain H238 was between 10 mg/L and 15 mg/L (Fig. 2b). In the control group without any rhamnolipid, the average OD_600_ value of strain *A. alternate* G2 was 2.640 So the half concentration (OD_600_) was 1.320. According to the inhibition curve in Fig. 2c, the IC50 value of mono-rhamnolipid against strain G2 was also lower than 5 mg/L, while the IC50 value of di-rhamnolipid against strain G2 was between 15 mg/L and 20 mg/L (Fig. 2d). Studies reported that the IC50 values of rhamnolipids products were ranged from 6 mg/L to 50 mg/L [27, 28]. The IC50 values can be used to compare the antimicrobial activity of mono-rhamnolipid and di-rhamnolipid in a more specific way. Results once again proved that the mono-rhamnolipid had better antimicrobial activity than di-rhamnolipid. The IC50 values can also guide the dosage of antimicrobial agents in the agricultural biocontrol applications.

### Antimicrobial mechanism of mono-rhamnolipid and di-rhamnolipid

**Fig. 3:**
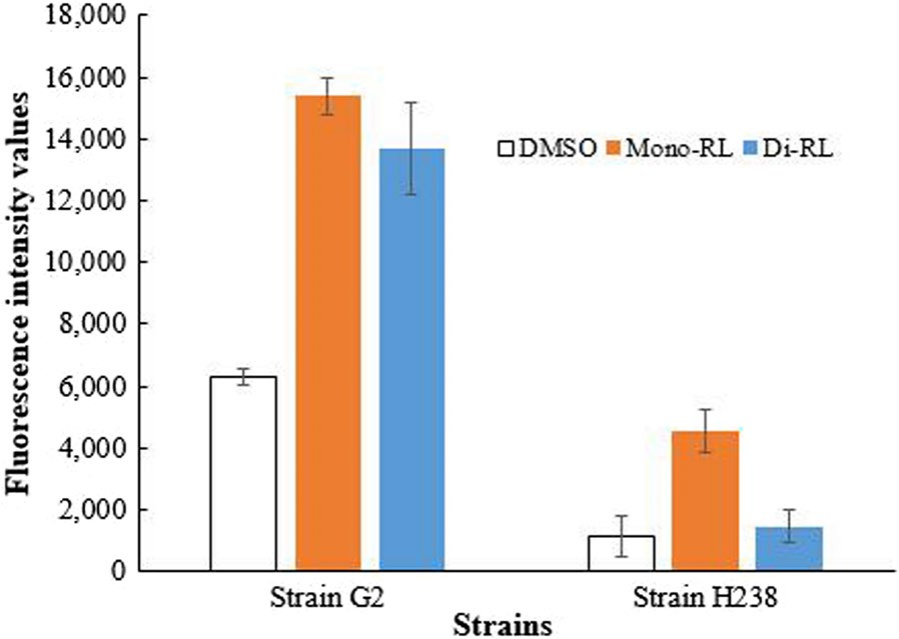
The fluorescence intensity after rhamnolipid stimulation indirectly indicating the intracellular ROS level: the control group of DMSO, environmental groups of mono-rhamnolipid (Mono-RL) and di-rhamnolipid (Di-RL)

As shown in Fig. 3, the fluorescence intensity in the groups of mono-rhamnolipid (Mono-RL) and di-rhamnolipid (Di-RL) was higher than in the control group of DMSO (*P* < 0.05). The fluorescence intensity in the group of Mono-RL was the highest. The fluorescence value was used as an indirect indicator of intracellular ROS level. Results demonstrated that rhamnolipid can lead to the accumulation of intracellular ROS in the tested strains. High ROS level can destroy nucleic acids and bioactive enzymes, and affect the growth and respiration metabolism microbial cells [29]. Due to containing one rhamnose, mono-rhamnolipid possesses better lipophilic properties. So mono-rhamnolipid is more likely to enter cells and cause cell damage of plant pathogens. The evoked ROS accumulation may be just one of antimicrobial mechanisms of rhamnolipid [30]. In this study, the ROS mechanism also confirmed the antimicrobial activity of mono-rhamnolipid was superior to that of di-rhamnolipid.

### Antimicrobial activity of rhamnolipid crude extracts

**Fig. 4:**
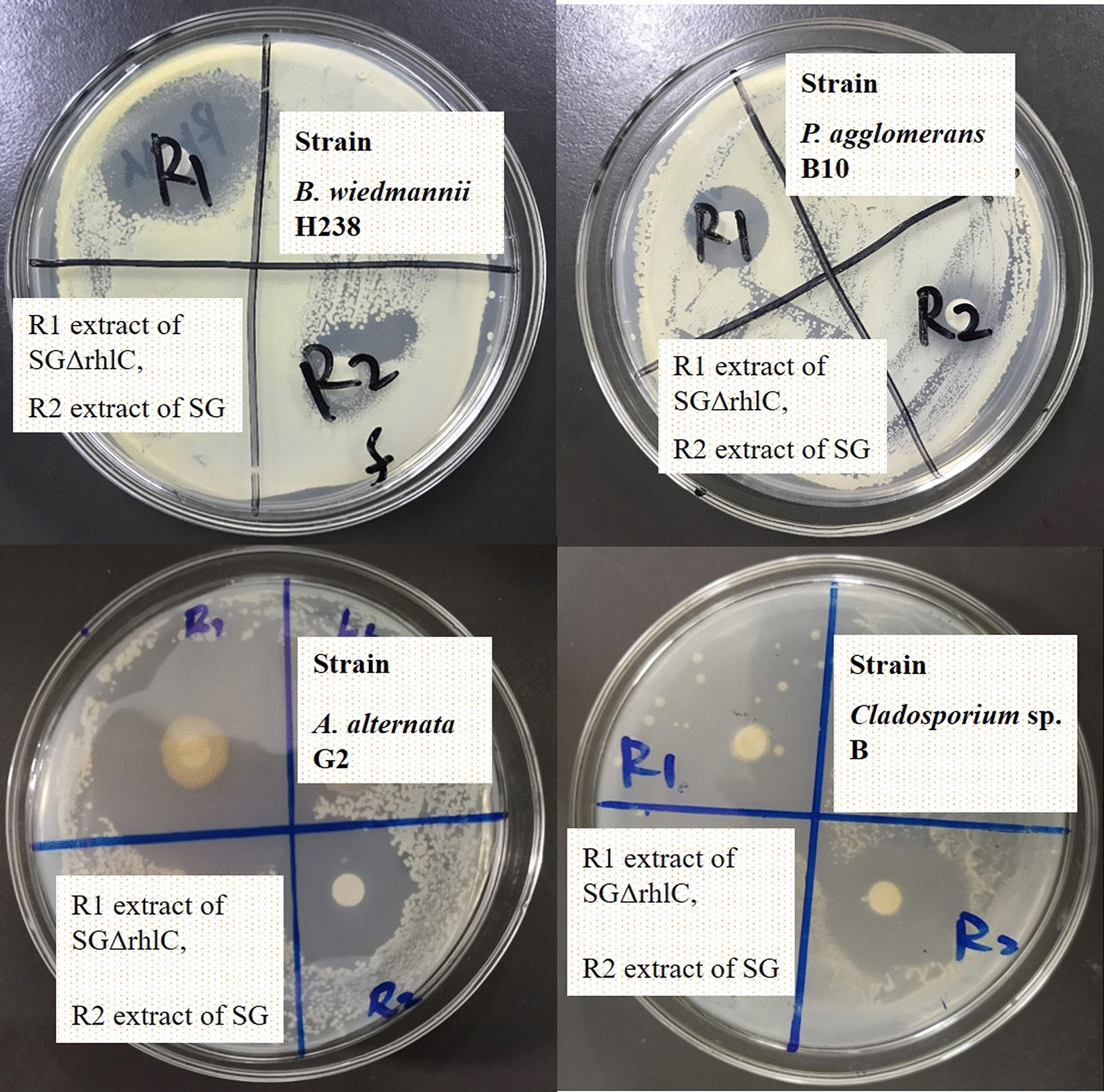
Inhibition zone of mono-rhamnolipid extract R1 from strain SGΔrhlC and mono/di-rhamnolipid extract R2 from strain SG

The mono-rhamnolipid extract R1 from the knockout strain SGΔrhlC and mono/di-rhamnolipid mixed extract R2 from wild-type strain SG were also used for antimicrobial activity evaluation. As shown in Fig. 4, both crude extracts R1 and R2 exhibited certain antimicrobial activity against the potential agricultural pathogens, such as *A. alternata*, *Cladosporium* sp. and *P. agglomerans*. According to the diameters of inhibition zones around filter papers, mono-rhamnolipid extract R1 showed better antimicrobial effect against all the 4 tested strains (*P* < 0.05). The diameters of inhibition zones formed by R1 against two fungi were larger than 30 mm. Results indicated that the mono-rhamnolipid crude extract R1 possessed antimicrobial activity with high efficiency and broad spectrum. The knockout strain *P. aeruginosa* SGΔrhlC is an excellent cell factory for high produce mono-rhamnolipid [17]. Therefore, it is possible to develop antimicrobial agents based on the mono-rhamnolipid crude extract produced by the knockout strain *P. aeruginosa* SGΔrhlC. It is a step closer to the economical antimicrobial agents for agricultural applications.

### Antimicrobial activity of the dilution of rhamnolipid fermentation broth

Mono-rhamnolipid (Mono-RL) is promising for agricultural biocontrol. The knockout strain *P. aeruginosa* SGΔrhlC can efficient produce mono-rhamnolipid [17]. In order to further reduce the application cost, the fermentation broth of strain *P. aeruginosa* SGΔrhlC was chosen to explore economical antimicrobial agents. The fermentation broth of strain SG was also comparatively investigated. As shown in Table 4, the fermentation broth of strain SGΔrhlC and strain SG also exhibited good antimicrobial activity after different times of dilution. Even after 50 times dilution, the diluted fermentation broth of the two strains had comparable antimicrobial effect with the rhamnolipid crude extracts R1 and R2 (concentration of 200 mg/L). The single factor analysis of variance was performed to compare the results of diluted fermentation broth. The diluted fermentation broth of strain SGΔrhlC showed better antimicrobial effect (*P* < 0.05). Because the main component in the fermentation broth of strain SGΔrhlC is mono-rhamnolipid. The results showed that the other substances in the fermentation broth did not affect the antimicrobial activity of rhamnolipid. *P. aeruginosa* is the most productive rhamnolipid-producing bacterium at present. The diluted fermentation broth of *P. aeruginosa* could be directly used as antimicrobial agent without rhamnolipid extraction. It can save time and costs in extraction and purification of rhamnolipid. And it is expected to achieve greater economic benefits in the agricultural field [31]. The cell-free culture broth of rhamnolipids was previously studied as a cost-effective fungicide against plant pathogens [32]. Diluted fermentation broth of the mono-rhamnolipid producing strain SGΔrhlC can be used as economical and effective agricultural antimicrobial agent.

**Table 4:** Inhibition rates of fermentation broth with different dilution ratio on microbial strains in liquid culture

Strains	Inhibition rate (%)							
	SGΔrhlC-10 times	SGΔrhlC-20 times	SGΔrhlC-50 times	R1	SG-10 times	SG-20 times	SG-50 times	R2
*E. coli* DH5α	35.5	33.1	33.8	30.8	28.8	28.1	28.4	16.3
*B. wiedmannii* H238	100.0	100.0	100.0	99.1	100.0	100.0	100.0	98.6
*B. safensis* B36#	100.0	100.0	100.0	100.0	100.0	100.0	100.0	99.2
*P. agglomerans* B10	33.7	31.5	29.5	31.3	19.6	18.7	18.98	14.9
*A. alternata* G2	97.2	97.2	97.1	97.1	96.8	96.4	96.52	96.6
*P. oxalicum* S11	96.8	96.6	96.4	95.1	94.4	93.9	94.10	93.6

## Discussion and perspectives

With the development of agriculture and the progress of science and technology, the agricultural pathogens control gradually tends to explore and apply more efficient, green, low toxicity and low residue antimicrobial agents. Long-term application of chemical agents in agriculture have brought problems such as microbial resistance, pesticide residue risk and environmental pollution [3–5]. Antimicrobial agents of biological origin are highly interested in control of agricultural pathogens.

Rhamnolipid is one of the most popular biosurfactants in research and application. In terms of agricultural applications, rhamnolipid can be used to improve soil properties, enhance the fertilizers efficiency and inhibit agricultural pathogens [8, 10]. Studies reported that rhamnolipid could control plant pathogen diseases, such as tomato blight, phytophthora capsicum, cucumber rot and sugarcane smut [33]. Rhamnolipid can inhibit microbial growth by changing cell permeability [34]. Rhamnolipid, as an agricultural antimicrobial agent, has the advantages of high activity, microbial source and wide antimicrobial spectrum.

*P. aeruginosa* is the most productive rhamnolipid-producing bacterium [11]. dTDP-L-rhamnose and β-hydroxy fatty acids are the two precursors for rhamnolipid synthesis. Rhamnolipids is divided into mono-rhamnolipid and di-rhamnolipid according to the contained rhamnosyl number. RhlB subunit of rhamnotransferase I is responsible for the synthesis of mono-rhamnolipid, and rhamnol transferase II (RhlC) catalyzes the synthesis of di-rhamnolipid [35].

In this study, the antimicrobial activity of mono-rhamnolipid and di-rhamnolipid were compared systematically. This work discovered that mono-rhamnolipid was superior to di-rhamnolipid in agricultural antimicrobial activity. Separation and purification of mono-rhamnolipid is too complex and high-cost. In order to explore economical antimicrobial agents, the mono-rhamnolipid fermentation broth of the knockout strain *P. aeruginosa* SGΔrhlC is a promising potential alternative. The knockout strain *P. aeruginosa* SGΔrhlC can produce 14.22 g/L mono-rhamnolipid [17]. Results showed that diluted fermentation broth of the mono-rhamnolipid producing strain SGΔrhlC can be used as economical and effective antimicrobial agent in agriculture.

In this study, mono-rhamnolipid exhibits important research and application value in the field of agricultural biocontrol. Mono-rhamnolipid has a wide antimicrobial spectrum and is microbial origin. Mono-rhamnolipid is an excellent candidate for the control of agricultural pathogens. Mono-rhamnolipid is expected to be developed as an efficient, green and broad-spectrum agricultural antimicrobial agent. It is simple, feasible, economical and effective to directly use the diluted fermentation broth of mono-rhamnolipid producing bacteria as agricultural antimicrobial agent. This study provided a new idea for efficient and green control of agricultural pathogens.

To further reduce the application cost of mono-rhamnolipid in agriculture, enhancing the production yield of mono-rhamnolipid is critical as well. Future research will be concentrate on breeding high mono-rhamnolipid producing strain and designing efficient fermentation process.

## Conclusions

Mono-rhamnolipid and di-rhamnolipid were successfully separated. Both mono-rhamnolipid and di-rhamnolipid exhibited antimicrobial activity to agricultural pathogens. But mono-rhamnolipid was superior to di-rhamnolipid. Mono-rhamnolipid possessed lower IC50 than that of di-rhamnolipid against both bacteria and fungi. ROS detection also confirmed mono-rhamnolipid with better antimicrobial activity. This work discovered that mono-rhamnolipid had greater potential in agricultural biocontrol. To explore economical antimicrobial agents, the mono-rhamnolipid producing strain *P. aeruginosa* SGΔrhlC is a promising alternative. Its mono-rhamnolipid crude extract and its diluted fermentation broth can be used as cost-effective agricultural antimicrobial agents. Results provided insights to develop green and efficient antimicrobial agents for agricultural applications.

## Data Availability

The datasets supporting the conclusions of this article are included within the article.
